# Porcine pancreatic ductal epithelial cells transformed with KRAS^G12D^ and SV40T are tumorigenic

**DOI:** 10.1038/s41598-021-92852-2

**Published:** 2021-06-28

**Authors:** Katie L. Bailey, Sara B. Cartwright, Neesha S. Patel, Neeley Remmers, Audrey J. Lazenby, Michael A. Hollingsworth, Mark A. Carlson

**Affiliations:** 1 Department of Surgery, University of Nebraska Medical Center, Omaha, NE 68198 USA; 2 VA Medical Center, Surgery 112, 4101 Woolworth Ave, Omaha, NE 68105 USA; 3 Department of Pathology and Microbiology, University of Nebraska Medical Center, Omaha, NE 68198 USA; 4 Eppley Institute for Research in Cancer, University of Nebraska Medical Center, Omaha, NE 68198 USA; 5 Fred & Pamela Buffett Cancer Center, University of Nebraska Medical Center, Omaha, NE 68198 USA; 6 Department of Genetics, Cell Biology and Anatomy, University of Nebraska Medical Center, Omaha, NE 68198 USA; 7 Center for Advanced Surgical Technology, University of Nebraska Medical Center, Omaha, NE 68198 USA

**Keywords:** Cancer, Cell biology

## Abstract

We describe our initial studies in the development of an orthotopic, genetically defined, large animal model of pancreatic cancer. Primary pancreatic epithelial cells were isolated from pancreatic duct of domestic pigs. A transformed cell line was generated from these primary cells with oncogenic KRAS and SV40T. The transformed cell lines outperformed the primary and SV40T immortalized cells in terms of proliferation, population doubling time, soft agar growth, transwell migration and invasion. The transformed cell line grew tumors when injected subcutaneously in nude mice, forming glandular structures and staining for epithelial markers. Future work will include implantation studies of these tumorigenic porcine pancreatic cell lines into the pancreas of allogeneic and autologous pigs. The resultant large animal model of pancreatic cancer could be utilized for preclinical research on diagnostic, interventional, and therapeutic technologies.

## Introduction

In the United States in 2020, approximately 57,600 people will be diagnosed with pancreatic cancer (~ 3.2% of all new cancer diagnoses), and there will be ~ 47,000 deaths from pancreatic cancer (~ 7.8% of all cancer deaths)^1^. The lifetime risk for pancreatic cancer is approximately 1 in 64^2^. The incidence of pancreatic cancer has been gradually increasing since the mid-1990s, and generally is higher in the African-American population^3^. Pancreatic cancer is now the fourth most common cause of cancer-related death in both men and women (after lung, prostate, and colorectal cancer, or lung, breast, and colorectal cancer, respectively)^1^. Despite advances in treatment modalities and strategies^4^, the mortality rate from pancreatic cancer has not decreased^1^. The overall 5-year survival rate from pancreatic cancer in the U.S. is 9%; 5-year survival rates in localized, regional (nodal spread), or metastatic disease were 37, 12, and 3%, respectively^1^. So, there remains a need for improved early diagnosis and therapy for pancreatic cancer.

Rodent models of pancreatic cancer may not accurately reflect human biology because of differences in anatomy, physiology, immune response, and genetic sequence between the two species^5–9^. Remarkably, only 5–8% of anti-cancer drugs that emerged from preclinical studies and entered clinical studies were ultimately approved for clinical use^10,11^. The cause of this low approval rate is multifactorial, but likely includes the less-than-optimal predictive ability of some murine models (e.g., tumor xenografting into immunosuppressed mice) to determine the efficacy of various therapeutics in humans^5–7,12–16^. Moreover, there are a number of genes for which the genotype–phenotype relationship is discordant between mice and human, including *CFTR*^–/–^ and *APC*^+/–17,^^18^. Incidentally, both the porcine *CFTR*^–/–^ and *APC*^+/–^ mutants reiterate the human phenotype (pulmonary/GI disease and rectal polyposis, respectively)^17–19^, in contradistinction to the murine mutants.

The recent trend to employ genetically engineered mouse models (GEMM), patient-derived xenografts (PDX), humanized mice, and in vivo site-directed CRISPR/Cas9 gene-edited mice in the testing of anti-cancer therapeutics may yield murine models with better predictive ability than obtained previously^7,20–24^. Though promising, these more advanced murine models come with increased cost and complexity^22^, and experience with them still is early. Importantly, all murine models have limited utility in the development of diagnostic or interventional technology that requires an animal subject whose size approximates a human. So, at present, there remains a need for improved animal models of pancreatic cancer that (1) are more predictive of human response to anti-cancer therapy^22,24^, and (2) are of adequate size for development of some diagnostic or interventional technologies. Herein we describe some first steps taken to develop an orthotopic porcine model of pancreatic ductal adenocarcinoma (PDAC) through genetic manipulation of key genes associated with PDAC within cultured porcine pancreatic ductal epithelial cells. Our intent with these initial experiments was to culture epithelial cells from the porcine pancreas and transform them into tumorigenic cell lines, which then could be used in future orthotopic implantation experiments in swine.

## Materials and methods

### Standards, rigor, reproducibility, and transparency

When feasible, the animal studies of this report were designed, performed, and reported in accordance with both the ARRIVE Guidelines (Animal Research: Reporting of In Vivo Experiments^25^) and the National Institutes of Health Principles and Guidelines for Reporting Preclinical Research^26,27^; for details, refer to Fig. S1 and Table S1, respectively.

### Materials, reagents, and animal subjects

All reagents were purchased through Thermo Fisher Scientific (www.thermofisher.com) unless otherwise noted. Short DNA sequences for vector construction, mutagenesis, and amplification purposes are shown in Table S2. Antibody information is given in Table S3. Athymic homozygous nude mice (Nu/J, Foxn1^nu^; females; 4 weeks old) were purchased from The Jackson Laboratory (www.jax.com). DNA sequencing was performed by the UNMC Genomics Core Facility (www.unmc.edu/vcr/cores/vcr-cores/genomics).

### Animal welfare

The animals utilized in this report were maintained and treated in accordance with the *Guide for the Care and Use of Laboratory Animals* (8th ed.) from the National Research Council and the National Institutes of Health^28^, and also in accordance with the Animal Welfare Act of the United States (U.S. Code 7, sections 2131–2159). The animal protocols pertaining to this manuscript were approved by the Institutional Animal Care and Use Committee (IACUC) of the University of Nebraska Medical Center (ID number 16‐133‐11‐FC). All procedures were performed in animal facilities approved by the Association for Assessment and Accreditation of Laboratory Animal Care International (AAALAC; www.aaalac.org) and by the Office of Laboratory Animal Welfare of the Public Health Service (grants.nih.gov/grants/olaw/olaw.htm). All surgical procedures were performed under isoflurane anesthesia, and all efforts were made to minimize suffering. Euthanasia was performed in accordance with the 2013 AVMA Guidelines for the Euthanasia of Animals^29^.

### Isolation of porcine pancreatic ductal epithelial cells

A detailed protocol for isolation of porcine pancreatic ductal epithelial cells (PDECs) is provided in Fig. S2. In brief, the intact pancreas from male domestic swine (age 5 mo) was harvested within 5 min after euthanasia, which was accomplished by transection of the intrathoracic inferior vena cava and exsanguination while under deep isoflurane anesthesia. The donor pigs had been on a separate research protocol (a study biomaterials within dorsal skin wounds), and had not received any recent medication other the anesthetics given for euthanasia; buprenorphine and cefovecin sodium had been given 4 weeks prior to euthanasia. Immediately after explantation of the pancreas, the main pancreatic duct was dissected sterilely with microsurgical instruments from the organ body, using 3.5 × loupe magnification. A 3–4 cm segment of proximal duct, from the duodenal ampulla to the mid-portion of the duodenal lobe of the porcine pancreas, was isolated and cleared of loose connective tissue. The duct was then minced with microscissors, and enzymatically digested in 10 mL of Collagenase I (10 mg/mL) plus DNase I (100 µg/mL) for 30 min at 37 °C, including mechanical disruption through a pipette tip after every 10 min of incubation. After 30 min the cells were pelleted (500 g × 5 min), the supernatant was discarded, and the cell pellet was resuspended in Epi Cell Growth Medium (ECM; Sigma, cat. no. 215–500) and pelleted a second time. The cells were then resuspended in 2 mL of complete medium, which was defined as ECM with 5% FBS and 1% Antibiotic–Antimycotic Solution (Corning Inc., cat. no. 30-004-CI), and incubated in a 6 well plates that were precoated with porcine gelatin (Corning Inc., cat. no. 354652). After 4 h the medium was transferred to a new well and 2 mL of fresh complete medium was added to the previous well. Epithelial colonies started to form after 5–7 days of culture under standard conditions (complete medium, 37 °C, 5% CO_2_). If fibroblasts were present on inverted phase microscopy, then light trypsinization was used to remove the contaminating fibroblasts. Light trypsinization consisted of a 3 min incubation with 1 mL of trypsin (Trypsin–EDTA 0.25%, Gibco™/ ThermoFisher, cat. no. 25200056), followed by washing with complete medium, and finishing with addition of 2 mL of the same. Standard trypsinization (detaching all cells for passaging) required a 10–15 min incubation. Cells were passaged in this fashion at least four times using 6-well plates, until no cells with fibroblast morphology were present. The resulting epithelial cells were passaged one time in 10 cm dishes prior to the below use.

### PDEC immortalization & KRAS vector

Primary PDECs were immortalized with SV40 large T antigen, using ready-to-use Lenti-SV40T (Puro) Lentivirus, High Titer cell immortalization kit (Applied Biological Materials, cat. no. LV613; www.abmgood.com), per the manufacturer’s instructions. The source of the porcine *KRAS*^G12D^ mutant was the plasmid used to generate the p53/KRAS Oncopig^30,31^. The *KRAS*^G12D^ cDNA was amplified out of this plasmid with primers (see Table S2) that flanked the sequence with *Xho*I and *Bam*HI restriction sites. The amplified product was inserted into the TOPO® vector (TOPO® TA Cloning® Kit; Invitrogen™/Life Technologies™, Thermo Fisher Scientific, cat. no. K202020) and verified by sequencing, as described above. The pLVX-IRES-ZsGreen1 (Takara Bio/ Clontech Laboratories, cat. no. 632187; www.takarabio.com) vector was cut with *Xho*I and *Bam*HI, and the *KRAS*^G12D^ sequence was ligated into this plasmid, producing a pLVX-IRES-ZsGreen1 vector which contained the mutant cDNA within its multiple cloning site (*KRAS*^G12D^ upstream).

The newly constructed plasmid, hereafter designated as LV-GK (LV- lentiviral; G = ZsGreen1; K = KRAS^G12D^), was transformed into One Shot™ Stbl3™ Chemically Competent *E. coli* (Invitrogen™/Thermo Fisher Scientific, cat. no. C737303), and plasmid DNA subsequently was isolated using a QIAGEN Plasmid Maxi Kit (cat. no. 12162), all per the manufacturer’s instructions. This plasmid then was transfected into Lenti-X™ 293 T cells, using Takara’s Lenti-X (VSV-G) Packing Single Shots (Takara Bio/ Clontech Laboratories, cat. no. 631275, www.takarabio.com; per the instructions), to generate infectious lentiviral particles that would produce direct expression of ZsGreen1 and KRAS^G12D^ mutant in the transduced cells. The medium for the Lenti-X™ 293 T cells was DMEM + 10% tetracycline-free FBS (Gemini Bio-Products, www.gembio.com, cat. no. 100–800).

### Cell transformations

Primary porcine pancreatic epithelial cells were grown to 80% confluency in 10 cm dishes under standard conditions, which generally required 14 days. The medium then was exchanged with supernatant from Lenti-X™ 293 T cells containing the LV-GK lentiviral particles, along with 1 µg/mL polybrene (cat. no. BM-862 M, Boston Bioproducts). After 24 h at 37 °C, fresh HEK293T (attc.org, cat. no. CRL3216) supernatant was added to the epithelial cells for a second incubation; after 48 h, the epithelial medium was changed to complete medium and incubated for an additional 24 h. The cells then were split into 6-well plates and cultured under standard conditions 80% confluency. The presence of transduced cells was determined with inverted fluorescent microscopy of living cells.

### Immunoblotting

Immunoblotting was performed to confirm expression of SV40T, decrease or loss of p53, and expression of KRAS^G12D^ (see Table S3 for a list of antibodies used), as previously described^32^. Immunoblot signal was detected using the Bio-Rad ChemiDoc™ MP Imaging System (bio-rad.com).

### Soft agar assay

A standard soft agar assay^33^ was used to determine anchorage-independent growth. A base layer of 1% agarose was plated into 6-well plates. A total of 20,000 cells/well were mixed with 0.7% agarose (750µL) and plated on top of the base layer. The plates were incubated under standard conditions for 12 days. The cells were then incubated with 200 µL of nitro blue tetrazolium chloride for 24 h to stain the colonies, and then counted using an inverted microscope. Mean count data were obtained from triplicate wells.

### Migration and invasion assay

Cells were incubated in serum-free medium for 24 h prior to plating the experiment. Basement membrane extract (BME, 1X; 100 µL; cat. no. 3455-096-02, R&D systems, rndsystems.com) was mixed with coating solution (1X; 100 µL; cat. no. 3455-096-03, R&D systems) was placed into Falcon™ Cell Culture Inserts (0.4 µm pore size; Falcon cat. no. 353095) and allowed to solidify overnight at 37 °C. The next day cells (50,000) were plated into the upper chamber (in triplicate) for both invasion (with BME) or migration analysis (without BME), and incubated under standard conditions for 48 h. The medium from the upper chamber then was aspirated away, and any cells remaining in the upper chamber were removed using a cotton swab. Cells that had migrated to the bottom of the membrane were stained with crystal violet and imaged.

### Population doubling assay

Cells were plated in 6-well plates (20,000 cell/well in 2 mL of complete medium; in triplicate) and cultured under standard conditions. Plates were trypsinized on days 3, 6, 9 and 12, and cells were counted with a hemocytometer. Cell number *vs*. day was plotted to determine the day range of linear growth. These data were used to determine population doubling time (DT) using the formula: DT = (∆t) × ln(2) ÷ ln(N_*f*_/N_*i*_) where ∆t = time interval between initial and final cell count, N_*f*_ = cell count at final time, and N_*i*_ = cell count at initial time^34^.

### Proliferation assay

Relative cell proliferation rates were determined using an MTT (3-(4,5-dimethylthiazol-2-yl)-2,5-diphenyltetrazolium bromide) assay kit (Vybrant™ MTT Cell Proliferation Assay Kit, Invitrogen™, Thermo Fisher Scientific, cat. no. V13154). Cells were plated in triplicate in a 96-well plate (3,000 cell/well in 200 µL of complete medium and cultured under standard conditions for 48 h. MTT reagent then was added to the cells per the manufacturer’s instructions, followed by addition of the solvent solution 3.5 h later. Absorbance was measured with a plate reader 3.5 h after solvent addition. Mean absorbance was normalized to absorbance from wild-type PDECs to calculate fold-difference in proliferation.

### Immunofluorescence and immunohistochemistry

Antibodies used in immunofluorescent and immunohistochemical experiments are listed in Table S3. Vector Laboratories (vectorlabs.com) ImmPress® goat (MP-7452) or rabbit (MP-7451) anti-mouse IgG polymer kits were used for IHC analyses per the manufacturer’s instructions. Detection of the HRP/ peroxidase enzyme was performed with SignalStain® DAB Substrate Kit from Cell Signaling (cat. no. 8059; www.cellsignal.com), per the manufacturer’s instructions.

### Subcutaneous tumorigenic cell injection

Subcutaneous implantation of tumorigenic cells was performed as previously described^35^, with some modifications. Transformed PDECs were trypsinized, counted, and resuspended in DMEM at a concentration of 5 × 10^6^ cells/mL. Nude mice (N = 10 per treatment group; 100% female; maintained in microisolator cages with soft bedding and fed regular chow ad lib) were allocated into treatment groups using an online randomization tool (www.randomizer.org). Using a 1 mL syringe with a 27-gauge needle, each subject was injected with a single dose of 500,000 cells in 100 µL of DMEM, into the subcutaneous space of the right hind flank without anesthetic. Tumors were allowed to grow for 4 weeks or until they reached 2 cm in diameter, as measured externally with a caliper, and then subjects were euthanized using an AVMA-approved^29^ method of CO_2_ asphyxiation. At necropsy all gross tumor was measured and collected, with samples undergoing formalin fixation, paraffin embedding, and H&E or immunohistochemical staining as described above. Sections were analyzed by an independent, unbiased (blinded to section identify) pathologist to determine tissue of origin and malignant features.

### Statistics and power analysis

Data are reported as mean ± standard deviation. Groups of continuous data were compared with ANOVA and the unpaired t-test. Categorical data were compared with the Fisher or Chi square test. For the power analysis of the murine subcutaneous tumor implant assay, tumor diameter was selected as the endpoint. Setting alpha to 0.05 and power (1 – beta) to 0.8, and with an estimated standard deviation of 20% of the mean, ten mice per group were needed across all treatment groups to detect a 30% difference in mean tumor diameter.

## Results

### Isolation of primary porcine PDECs

**Figure 1:**
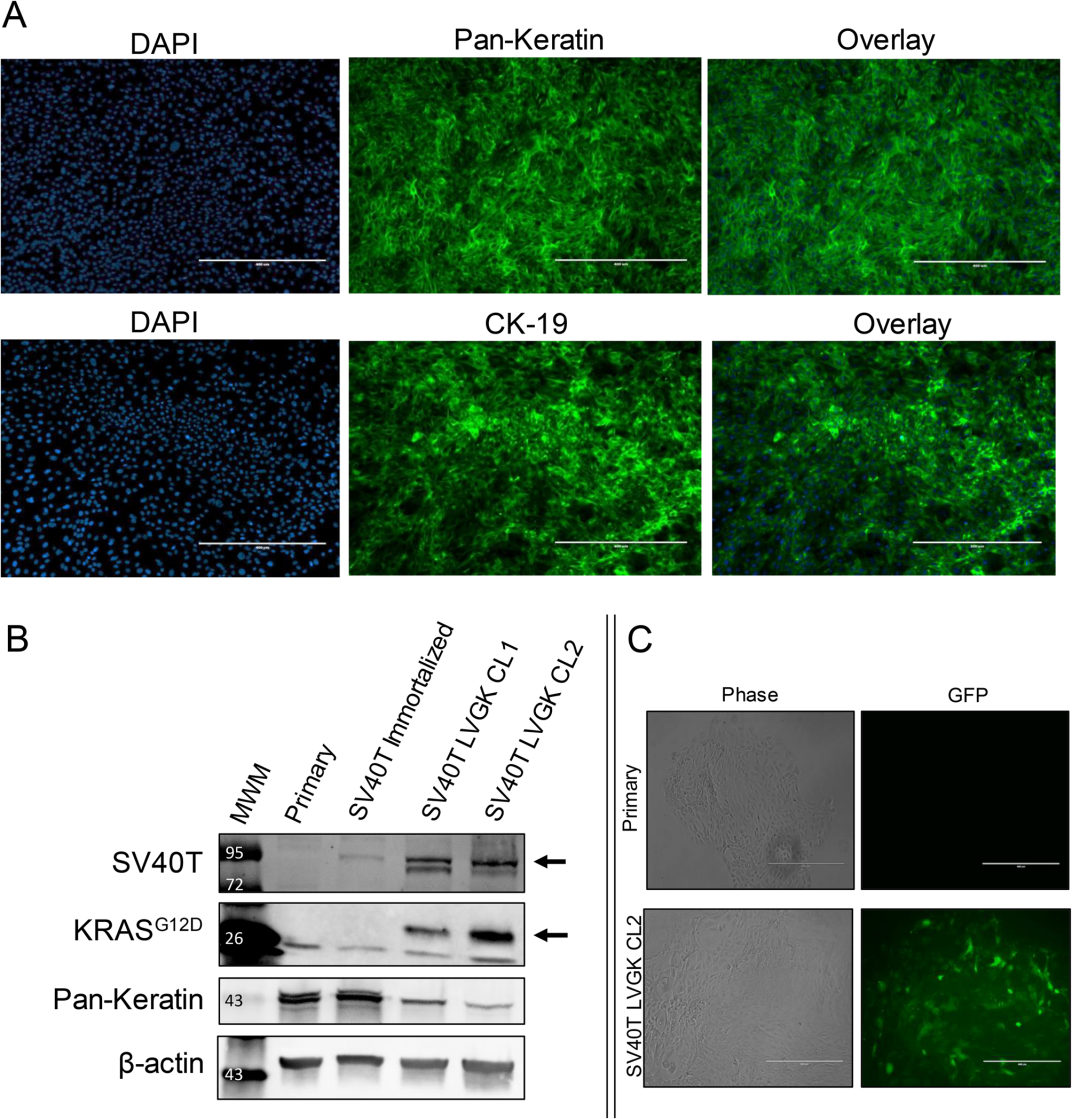
Isolation and transduction of primary porcine pancreatic ductal epithelial cells. (**A**) Immunofluorescent staining for Pan-Keratin and cytokeratin 19 (CK-19) in cultured primary epithelial cells. (**B**) Immunoblot for SV40T, mutant KRAS^G12D^, and Pan-Keratin in primary epithelial cells, SV40T immortalized cells, and SV40T immortalized cells transformed with KRAS^G12D^ (SV40T LV-GK; two lines, CL1 and CL2) Samples are from the same gel/blot with different IR Dye exposures depending on the secondary for the primary antibody. For full blot images see Fig. S3. Black arrow = band of interest; MWM = molecular weight marker. (**C**) Expression of ZsGreen1 (a green fluorescent protein variant) in primary epithelial cells and SV40T immortalized cells transduced with the LV-GK; phase and inverted fluorescent microscopy of living cells. Measure bars = 400 µm.

Cells cultured from microdissected porcine pancreatic ducts displayed epithelial morphology under phase microscopy (not shown) and stained for CK19 and Pan-Keratin (markers of pancreatic ductal epithelium^36^; Fig. 1A). These data supported the conclusion that we had isolated primary porcine pancreatic ductal epithelial cells (PDECs), on which we could proceed performing the immortalization and transformation.

### Generation of transformed PDEC lines

In order to transform our primary PDECs, we first immortalized the cells with SV40 large T antigen (SV40T) to inhibit endogenous p53. Inactivating mutations of the tumor suppressor p53 protein occur in approximately 50–75% of PDAC cases^37–39^. SV40T binds to the same domain of p53 where missense mutations occur, and mimics the effect seen with these mutations^40,41^. Expression of SV40T after lentiviral insertion was confirmed with immunoblotting (Fig. 1B and S3). We also determined p53 and p21 expression levels (Fig. S4) to confirm downregulation of p53 and p21 with SV40T expression^42^.

We then generated a lentiviral vector containing the porcine *KRAS*^G12D^ sequence previously identified^30^ as the porcine equivalent to the mutant *KRAS* which is present in multiple human cancers^38,43–46^; expression of the murine version of this mutant KRAS was the basis for the KRAS/p53 (KPC) mouse, a genetically engineered murine model of pancreatic cancer^47^. We utilized a vector (Fig. S5) that contained the human cytomegalovirus immediate early promoter that is upstream from a multiple cloning site, which allows for both the gene of interest and a fluorescent protein marker (ZsGreen1^48^) to be translated in a single bicistronic mRNA. Expression of KRAS^G12D^ after lentiviral insertion was confirmed with immunoblotting and fluorescent microscopy of living cells (Fig. 1B,C and S3).

We also observed decreased Pan-Keratin (Fig. 1B and S3) and E-cadherin (Fig. S4) expression in the transformed cell lines which has been observed in other PDAC cell lines, and has been associated with epithelial to mesenchymal transition during neoplastic invasion, growth, and metastasis^49,50^. Over time, expression of E-cadherin can decrease, as has been shown in some human pancreatic cancer samples^51,52^. Based on all of the above expressional data, we concluded that the generated cell lines had been transformed with KRAS^G12D^ and SV40T.

### In vitro behavior of transformed PDEC lines

**Figure 2:**
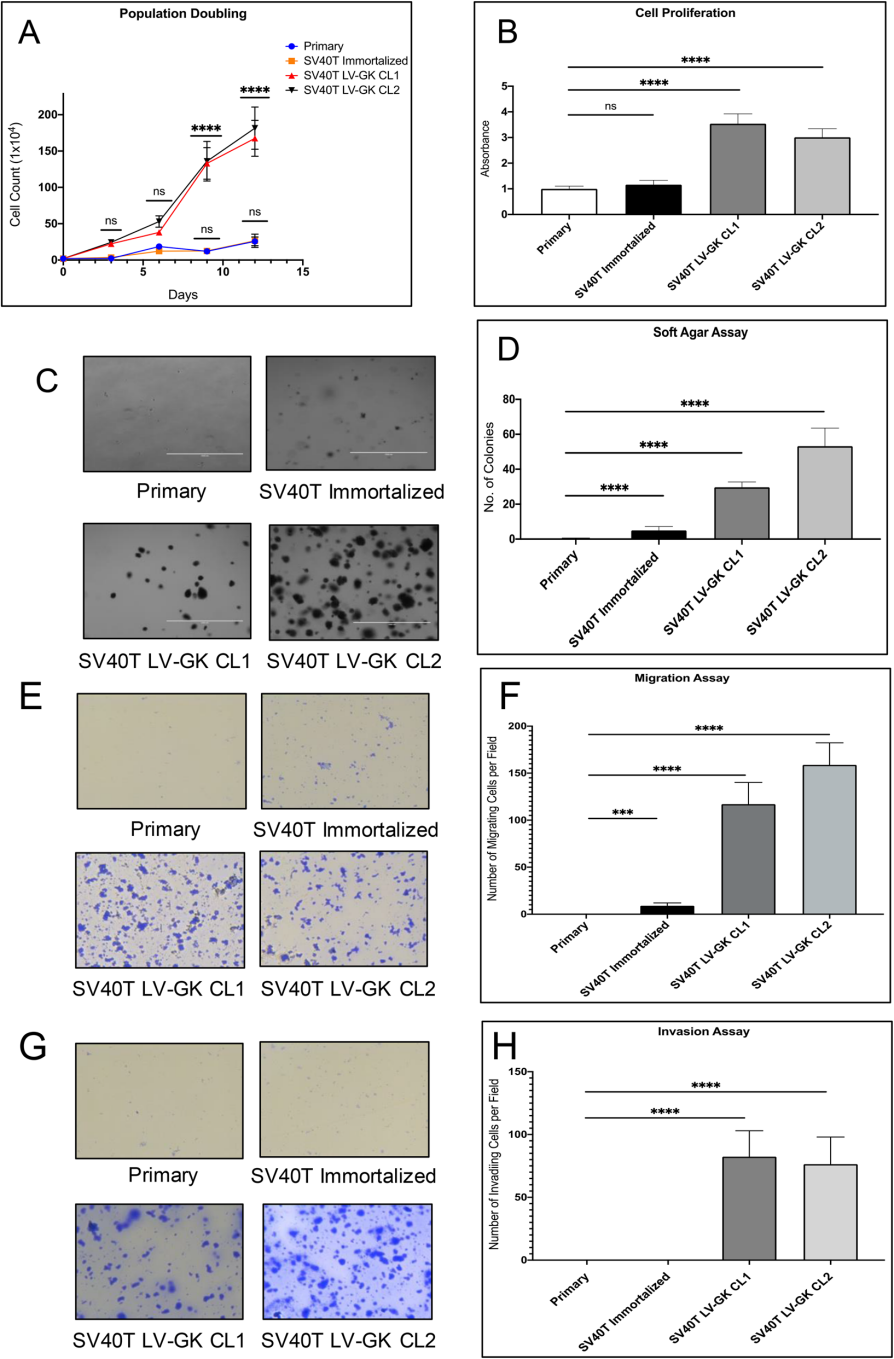
In vitro cell transformation assays. Cultured primary epithelial cells from porcine pancreatic duct were compared with SV40T immortalized and transformed (SV40T + KRAS^G12D^) cell lines. (**A**) Cell culture population doubling time (count-based analysis). (**B**) Proliferation rate using metabolic dye assay, represented as fold change with respect to primary cells (defined as 1). (**C**) Phase contrast images of soft agar assay (bar = 1000 µm). (**D**) Plot of soft agar assay. (**E**–**F**) Transwell migration assay (absence of BME), phase images and plot. (**G**–**H**) Transwell invasion assay (presence of BME), phase images and plot. Each bar or data point in this Figure represents the mean ± SD of triplicate wells; each experiment performed three times on separate days (one representative experiment shown in each panel); ****p < 0.0001; ns = not significant (p ≥ 0.05).

Two lines of transformed PDECs (SV40T LV-GK CL1 and SV40T LV-GK CL2, abbreviated as CL1 and CL2) were tested in a series of in vitro transformation assays. Population doubling time was shorter and cellular proliferation (metabolic dye conversion) was greater for both CL1 and CL2 compared to primary and SV40T immortalized cells (Fig. 2A–B). Calculation from the data in Fig. 2A demonstrated that the doubling time for the transformed cell lines was approximately the same at ~ 15 h, compared to the ~ 3 d doubling time of primary and SV40T cells (a ~ fivefold difference). Both CL1 and CL2 demonstrated increased (≥ tenfold) soft agar colony formation over primary and SV40T immortalized cells (Fig. 2C–D), as well as increased migration and invasion capability compared to primary and SV40T immortalized cells (Fig. 2E–H). Based on these in vitro assays of transformation, we suspected that both our transformed cell lines had the potential to form tumors in vivo. With the exception of higher soft agar colony formation in CL2 (Fig. 2C–D), we were unable to demonstrate an obvious difference in transformed behavior between CL1 and CL2. Based on the difference in the soft agar colony assay, we elected to utilize CL2 in the below in vivo tumorigenicity assay.

### Transformed PDECs generated subcutaneous tumors with PDAC features when injected into immunodeficient mice

**Figure 3:**
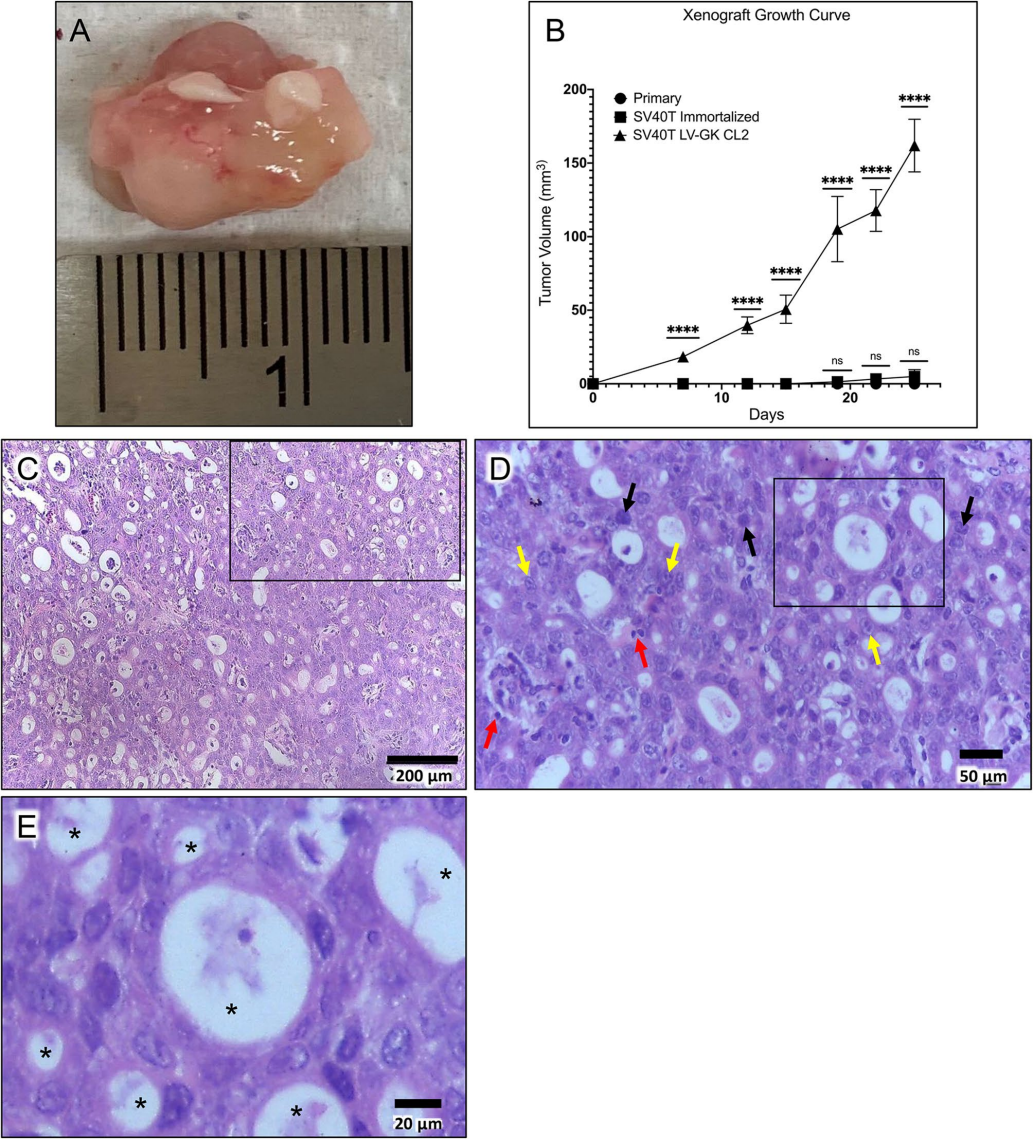
In vivo tumorigenesis assay. (**A**) Representative xenograft tumor explanted from a nude mouse 42 days after subcutaneous injection of transformed epithelial cells (SV40T LV-GK CL2). (**B**) Comparison of xenograft tumor volume between primary epithelial cells versus the SV40T immortalized and SV40T LV-GK CL2 cell lines; ****p < 0.0001; ns = not significant (p ≥ 0.05). (**C**) H&E microscopic image of xenograft tumor. (**D**) Higher power H&E image; region of interest box shown in panel C. Black arrows = hyperchromatic nuclei; yellow arrows = nuclei with large nucleoli; red arrows = pyknotic nuclei. (**E**) Higher power H&E image; region of interest box shown in panel D. *Asterisks indicate lumen of gland-like structures lined with ductal-like cells; lumen were filled with mucin in the Alcian blue staining (Fig. S6).

In order to determine if transformed PDECs were tumorigenic in vivo, we utilized homozygous athymic model of subcutaneous cell implantation (xenografting into nude mice)^16^. Primary, immortalized SV40T and transformed SV40T LV-GK CL2 cells were injected into the right flank of nude mice (500 K cells/site; 1 site/mouse). Neither the primary nor the immortalized SV40T cells grew tumors. The transformed cell line grew sizeable tumors (~ 150 mm^3^) within 4 weeks (Fig. 3A–B). The subcutaneous tumors were vascularized and mucinous in gross appearance (Fig. 3A). Alcian blue staining confirmed this phenotypical observation by showing mucin staining within small glands (20–50 µm diameter) within the tumor microenvironment (Fig. S6), more consistent with PDAC and less with cystic neoplastic disease.

Pathological review of H&E staining from tumor xenografts demonstrated that the tumor had architectural features of an adenocarcinoma^53^ with formation of back-to-back glandular lumens, the so-called cribriform pattern (Fig. 3C,D). The nuclei in the carcinomatous glands had multiple anaplastic features, including pleomorphism with a wide variation in size and shape of nuclei, with abnormal nuclear morphology including hyperchromasia and pyknosis (Fig. 3D). As is typical for an adenocarcinoma, the malignant glands produce mucin which is visible in the lumen of the large central gland on H&E stains (Fig. 3D,E), and as was seen with the Alcian blue staining (Fig. S6). There also were occasional large nucleoli (another anaplastic feature) seen at higher power (Fig. 3D).

The tumors also stained for collagen deposition to a variable degree, as determined with trichrome staining (Fig. S7). Collagen deposition is an extensively studied component of tumor-associated stroma in PDAC, and is associated with tumor progression and invasion^54^. These findings supported our conclusion that we had generated xenograft tumors which had some phenotypic features of human PDAC.

### Immunohistochemical features of tumor xenografts

**Figure 4:**
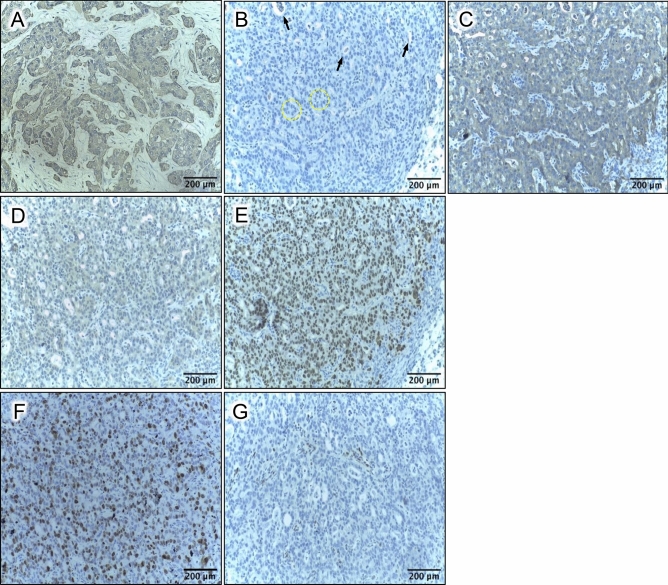
Immunohistochemistry of tumor xenografts from subcutaneous nude mouse assay. (**A**) Pan-Keratin; section from human PDAC (original tumor, not a xenograft); utilized as a positive control (nuclear counterstain = hematoxylin). Panels B-G are all from xenograft tumor, obtained with implantation of the SV40T LV-GK CL2 cell line. (**B**) Negative control (all reagents except no primary antibody). Black arrows = ductal-like structures; dashed yellow circles = acinar-like structures. (**C**) Pan-Keratin. (**D**) KRAS^G12D^ (antibody specific to mutant KRAS). (**E**) SV40T. (**F**) Ki-67. (**G**) p21.

Tumor xenografts from the subcutaneous implant model underwent immunohistochemical staining with Pan-Keratin, E-cadherin, SV40T, KRAS^G12D^, p21, and Ki-67 (Fig. 4 and S8). Tumors stained positive for both Pan-Keratin and E-cadherin. As further confirmation of ductal phenotype, the xenografts also stained positive for carbonic anhydrase (Fig. S8). All tumor xenografts formed ductal and acinar-like structures (similar to what is seen in human PDAC; Fig. 4A–C), consistent with a tumor of ductal and/or alveolar origin. Confirmation of lentiviral transduction was demonstrated with KRAS^G12D^ and SV40T staining (Fig. 4D–E). These tumors also were positive for Ki-67 (Fig. 4F) and PCNA (Fig. S9), which are both proliferative markers that are expressed in human PDAC and correlate with poor prognosis^55,56^. Tumor cells did not stain for p21 expression, with the exception of some infiltrating lymphocytes, consistent with loss of p53 function with SV40T expression in the tumor cells (Fig. 4G). Positive control staining for p21 in the skin of these immunodeficient mice is shown in Fig. S10.

### Preliminary orthotopic implantation in wild type swine

A next logical step in development of a new large animal orthotopic model of PDAC would be to implant the transformed PDEC cells into the pancreas of a mature wild type swine. Though this was not the goal with the present manuscript, we have begun to perform these experiments, and have shown some preliminary data of such an implantation in Fig. S11. The pancreas of two wild type minipigs (~ 80 kg) was focally injected with 40 million transformed PDECs (SV40T LVGK CL2); see Fig. S11 for methodology. At necropsy 8 weeks later, there were no gross abnormalities. However, at each injection site there were microscopic (up to ~ 800 µm) circumscribed abnormalities which appeared to the ductal metaplasia on H&E (possibly a response to injury), with cords of surrounding T-lymphocytes (Fig. S11D,H). Interestingly, immunohistochemistry for KRAS^G12D^ was positive in this abnormality, but negative for SV40T (Fig. S11F,G). It was not clear whether this circumscribed abnormality represented an evolving tumor, an involuting collection of injected cells, an endogenous response to injury, or something else. But expression of at least one of the transgenes was evident. Of note, the role of the immune response against this allogeneic cellular implant has not yet been characterized (studies are pending).

## Discussion

Porcine biomedical models have been used for decades in the fields of trauma and hemostasis^57^, xenotransplantation^58,59^, dermal healing^60^, toxicology^61^, atherosclerosis^62^, and cardiac regeneration^63^; the utility of these models is growing. A porcine genome map was generated in 2012^64^, and further coverage, annotation, and confirmation is ongoing^65,66^. Porcine-centered online tools and databases are now available^67^. Genetic manipulation of pigs (including knockouts, tissue-specific transgenics, inducible expression^30,68–75^) with similar tools as used in the mouse is becoming more routine, with new gene-edited porcine models emerging for diseases such as atherosclerosis, cystic fibrosis, Duchenne muscular dystrophy, and ataxia telangiectasia^76,77^.

The rationale to build a porcine model of pancreatic cancer is (1) to have a platform for diagnostic/therapeutic device development otherwise not achievable in murine models; and (2) to have a highly predictive preclinical model in which anti-cancer therapies (including immunotherapies) could be vetted/optimized prior to a clinical trial^78^. The rationale to use the pig in this modeling effort is that this species mimics human genomics^65,79–82^, epigenetics^83^, physiology^61,79,84,85^, metabolism^79,85,86^, inflammation and immune response^82,87–91^, and size^85,92^ remarkably well (in particular, better than mice), with reasonable compromises towards cost and husbandry^85^. So, based on the pig’s relatively large size and its proven track record in replicating human biology which, incidentally, is a demonstrably better replication than can be obtained with rodents, we selected swine as the model organism for this pancreatic cancer project.

Research on immunocompetent large animal cancer models^93–95^ includes prostate cancer, for which there is a canine model^96^. In addition, Munich investigators reported the engineering of (1) an *APC* mutant pig that developed rectal polyposis^17,97^ and (2) a pig with Cre-inducible p53 deficiency^73^. This group subsequently determined that their p53-null subjects (*TP53*^R167H/R167H^) developed osteosarcoma by age 7–8 months^98^. Other p53-deficient pigs have been engineered since this initial report^74,99^; in the report from Iowa, half (5 out of 10) of p53-deficient (*TP53*^R167H/R167H^) pigs developed lymphoma or osteogenic tumor at age 6–18 months^74^. A group in Denmark reported the creation of a *BRCA* mutant pig in 2012^100^. In 2017, another genetic porcine model of intestinal neoplasia was reported^101^, utilizing inducible expression of KRAS^G12D^, c-Myc, SV40 large T antigen, and retinoblastoma protein (pRb).

A KRAS/p53 “Oncopig” was reported in 2015^31,94^. This subject has a somatic LSL-cassette that can express dominant negative p53 (R167H mutation) and activated KRAS (G12D mutation); i.e., the porcine analog of the KRAS/p53 mouse^47,102^. Site-specific expression of Cre recombinase in the Oncopig resulted in localized p53 inhibition and KRAS activation; subcutaneous injection of AdCre produced mesenchymal tumors at the injection sites^31^ in non-immunosuppressed Oncopigs.

In 2018, induction of autochthonous pancreatic tumor in one KRAS/p53 Oncopig was accomplished^103^ by injection of adenovirus-expressing-Cre into the pancreatic duct, in order to minimize transformation of non-epithelial cells. At the 12-month time point in this single subject, pancreatic tumor was not evident radiographically nor grossly, but was visible after organ sectioning. We recently posted our preliminary data on the induction of pancreatic cancer in 14 Oncopig subjects^107^, in which we observed fulminant, grossly evident pancreatic tumor growth in 10 subjects within 2 weeks of AdCre injection into the duct a surgically-isolated pancreatic lobe. In 2017, initial work was published on a Oncopig-based model of hepatocellular carcinoma^104^.

Löhr et al.^105^ demonstrated that bovine pancreatic ductal cells could be transformed with SV40T and mutant KRAS. They orthotopically implanted these transformed cells into nude mice, and observed pancreatic tumor development and metastasis to the liver. Adam et al.^30^ transformed primary porcine dermal fibroblasts with retroviral insertion of hTERT, p53^DD^ (dominant negative), cyclin D1, CDK4^R24C^, c-Myc^T58A^ and H-Ras^G12V^. These transformed cells also grew tumors after subcutaneous injection in both immunodeficient mice and autologous wild type swine. Of note, the latter required immunosuppression with cyclosporine, prednisone, and azathioprine to prevent the host from rejecting the implanted cells. Our work differs from the previous publications in that we created tumorigenic cell lines by transforming ductal epithelial cells obtained from the porcine pancreas which, similar to bovine pancreatic ductal cells, only required KRAS activation and p53 inhibition.

There are alternative approaches for a orthotopic porcine model of PDAC. The Oncopig^31^ can generate PDAC^103,106,107^ in vivo, but this model contains a somatic LSL-cassette that is present in every cell, so off-target transformation is possible. This off-target effect can result in transformation of multiple cell types and pleomorphic tumors^107^. Secondary to this poor specificity, deriving cell lines from tumors induced in vivo within the Oncopig pancreas would be a less desirable approach for the orthotopic porcine model. Isolation of primary pancreatic ductal epithelial cells from the Oncopig pancreas with subsequent transformation in vitro in order to generate a tumorigenic cell line would be a reasonable alternative approach to KRAS/SV40T approach of the present report. However, the Oncopig approach would be more expensive than using wild type pigs. In addition, use of Oncopig tissue for starting genetic material limits the selection of indel targets; i.e., the Oncopig is obligated to express KRAS^G12D^ and p53^R167H^ after transformation. The investigator can select indel targets as desired when working with wild type tissue. Whether or not an Oncopig-derived cell line would be superior to the KRAS/SV40T cell line of the present report for mimicking PDAC in pigs is not known. So, the advantages of using wild type pancreatic tissue over Oncopig tissue to generate transformed cell lines for orthotopic implantation include (1) less cost and (2) flexibility in the selection of indel targets.

Our goal with this project was to generate tumorigenic pancreatic cell lines that could be used in an immunocompetent porcine model of pancreatic cancer. Murine models may be limited in their ability to replicate human biology and size, so a large animal model of pancreatic cancer might enhance our ability to develop and test new diagnostic and treatment modalities for this disease. The data presented herein demonstrated that wild type porcine pancreatic ductal epithelium can be transformed with SV40T and mutant KRAS^G12D^; these transformed cells subsequently can grow tumors in immunodeficient mice, displaying histological and phenotypical features similar to human PDAC. These data provide a pathway for the construction of an orthotopic porcine model of pancreatic cancer, namely, implantation of tumorigenic pancreatic epithelial cells into the pancreas. Future work will focus on orthotopic implantation studies in pigs, in order to define optimal implantation conditions, describe the host immune response, and provide model characterization and validation.

## Data availability

Datasets from this manuscript will be freely shared upon request to the senior author (MAC).

## Supplementary Information

Supplementary Information.
